# Tetra­aqua­(ethane-1,2-di­amine-κ^2^ *N*,*N*′)nickel(II) naphthalene-1,5-di­sulfonate dihydrate

**DOI:** 10.1107/S2414314623010325

**Published:** 2023-12-14

**Authors:** Jabbor R. Suyunov, Khayit Kh. Turaev, Bekmurod Kh. Alimnazarov, Aziz B. Ibragimov, Islombek J. Mengnorov, Abdusamat A. Rasulov, Jamshid M. Ashurov

**Affiliations:** a Termez State University, Barkamol Avlod Street 43, Termez City, Uzbekistan; b Institute of General and Inorganic Chemistry of Uzbekistan Academy of Sciences, 100170, Mirzo Ulug’bek Str. 77a, Tashkent, Uzbekistan; c Institute of Bioorganic Chemistry, Academy of Sciences of Uzbekistan, 100125, M. Ulugbek Str. 83, Tashkent, Uzbekistan; Vienna University of Technology, Austria

**Keywords:** ethane-1,2-di­amine, naphthalene-1,5-di­sulfonate, crystal structure, hydrogen bonding, C—H⋯π inter­action

## Abstract

The reaction of ethane-1,2-di­amine (en, C_2_H_8_N_2_), the sodium salt of naphthalene-1,5-di­sulfonic acid (H_2_NDS, C_10_H_8_O_6_S_2_), and nickel sulfate in an aqueous solution resulted in the formation of the title salt, [Ni(C_2_H_8_N_2_)(H_2_O)_4_](C_10_H_6_O_6_S_2_)·2H_2_O or [Ni(en)(H_2_O)_4_](NDS)·2H_2_O. In the asymmetric unit, one half of an [Ni(en)(H_2_O)_4_]^2+^ cation and one half of an NDS^2−^ anion, and one water mol­ecule of crystallization are present. The Ni^2+^ cation in the complex is positioned on a twofold rotation axis and exhibits a slight tetra­gonal distortion of the *cis*-NiO_4_N_2_ octa­hedron, with an Ni—N bond length of 2.0782 (16) Å, and Ni—O bond lengths of 2.1170 (13) Å and 2.0648 (14) Å. The anion is completed by inversion symmetry. In the extended structure, the cations, anions, and non-coordinating water mol­ecules are connected by inter­molecular N—H⋯O and O—H⋯O hydrogen bonding, as well as C—H⋯π inter­actions, forming a three-dimensional network.

## Abstract 2

In the title salt, [Ni(en)(H_2_O)_4_]^2+^·NDS^2−^·2H_2_O, the Ni^2+^ cation is positioned on a twofold rotation axis and exhibits a coordination number of six. The components are connected through an extensive network of O—H⋯O and N—H⋯O hydrogen bonds, and C—H⋯π inter­actions.

## Graphical abstract

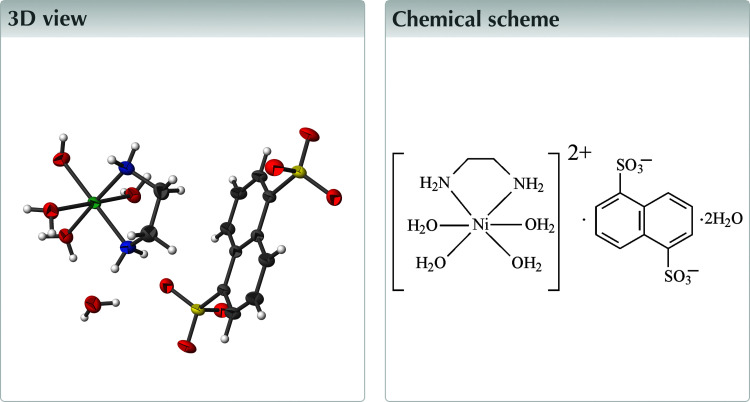

## Structure description

As known for decades, ethane-1,2-di­amine (en) exhibits excellent coordination and chelating abilities, forming five-membered rings with the central metals. Generally, these metallacycles adopt a twist conformation. En ligands can coordinate with metal ions in a monodentate fashion (Xue *et al.*, 2016 ▸; Mitzinger *et al.*, 2016 ▸) and in some complexes, they can act as bridging ligands (Binnemans *et al.*, 2013 ▸; Bratsos *et al.*, 2011 ▸; Doring & Jones, 2013 ▸). In most cases, en demonstrates chelating properties (Ashurov *et al.*, 2018 ▸; Qadir *et al.*, 2020 ▸). There are also metal complexes where non-coordinating en mol­ecules are present (Sun *et al.*, 2017 ▸; Tian *et al.*, 2017 ▸; Mirzaei *et al.*, 2014 ▸).

Complexes derived from naphthalene-1,5-di­sulfonic acid (H_2_NDS) are of great inter­est in supra­molecular chemistry due to their ability to form hydrogen bonds (Shi *et al.*, 2014 ▸; Xu *et al.*, 2019 ▸; Chen *et al.*, 2020 ▸; Suyunov *et al.*, 2023 ▸), because the sulfonate group can accept up to six hydrogen bonds with its lone pairs (Oh *et al.*, 2020 ▸; Chen *et al.*, 2022 ▸). As a ligand, NDS^2−^ sometimes binds in a bridging mode (Lian & Qu, 2013 ▸; Das *et al.*, 2015 ▸; Tai *et al.*, 2015 ▸). As part of our work in this area, we now describe the synthesis and structure of the hydrated title salt [Ni(en)(H_2_O)_4_]^+^·NDS^2−^·2H_2_O.

**Figure 1:**
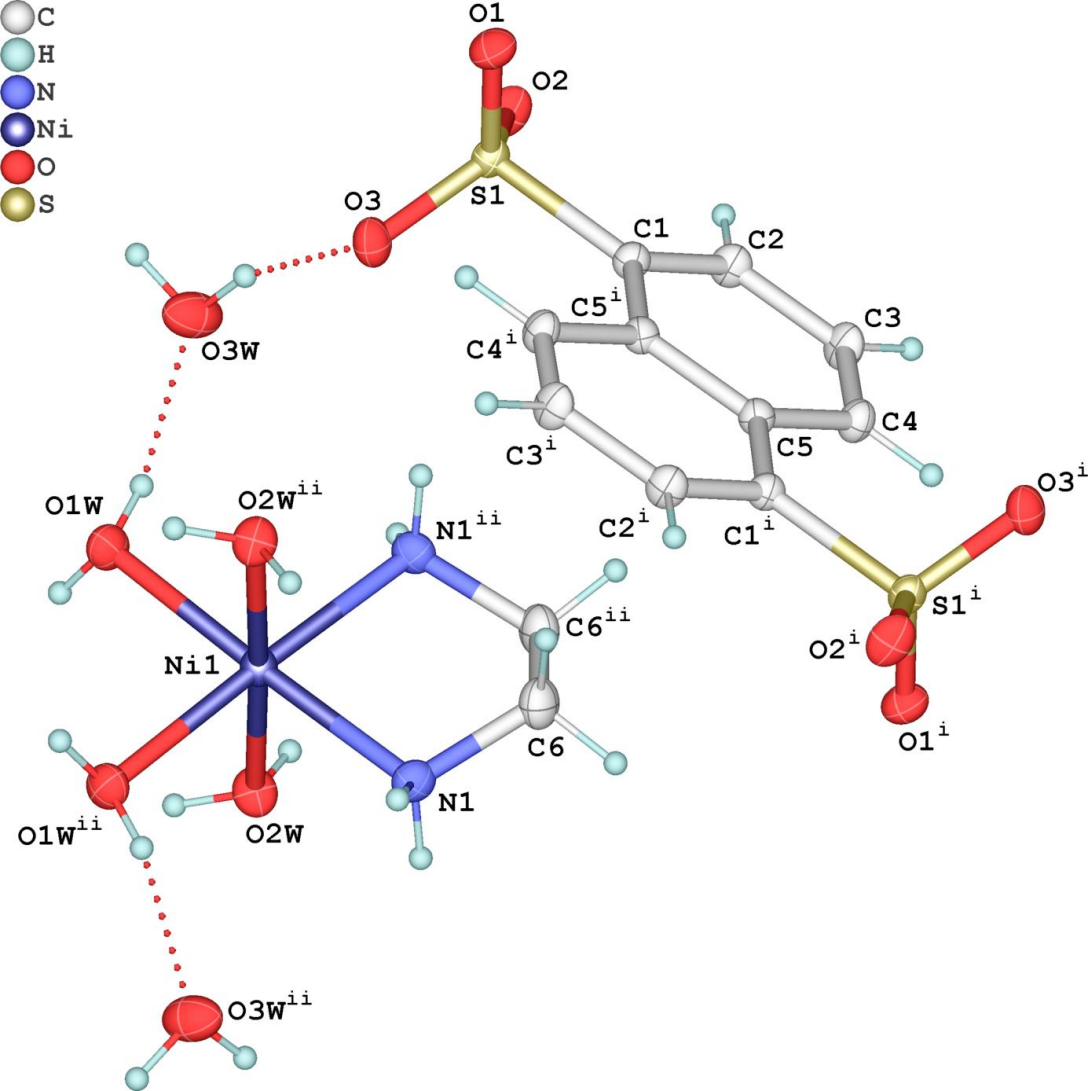
The structures of the mol­ecular entities in the title salt, showing the atom-labeling scheme and displacement ellipsoids drawn at the 50% probability level. H atoms are shown as spheres of arbitrary radius and hydrogen bonds are shown as dashed lines. [Symmetry codes: (i) 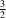 − *x*, 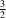 − *y*, 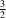 − *z*; (ii) 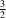 − *x*, *y*, 1 − *z*.]

The asymmetric unit consists of one-half of the [Ni(en)(H_2_O)_4_]^2+^ complex cation, one half of the NDS^2−^ organic dianion, and a water mol­ecule of crystallization. The Ni^2+^ cation in the complex is positioned on a twofold rotation axis and exhibits a slightly tetra­gonal distortion of the *cis*-NiO_4_N_2_ octa­hedron. The Ni—N bond length is 2.0782 (16) Å, and the Ni—O bond lengths are 2.1170 (13) Å and 2.0648 (14) Å, similar to those reported for other [Ni(en)(H_2_O)_4_]^2+^ complexes (Healy *et al.*, 1984 ▸). The en ligand conformation conforms to the crystallographic twofold axis that passes through it. The NDS^2−^ dianion exhibits inversion symmetry, with the inversion center located at the middle point of the C5—C5( 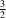 − *x*, 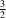 − *y*, 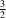 − *z*) bond. The structures of the mol­ecular entities are shown in Fig. 1 ▸. Neighboring anions have two distinct orientations relative to the complex cation, with the angle between their planes being 55.06 (7)°. The naphthalene ring system exhibits typical bond lengths and angles, with C—C bond lengths ranging from 1.368 (2) to 1.429 (2) Å, and C—C—C angles within the range 117.98 (18) to 123.08 (15)°.

**Figure 2:**
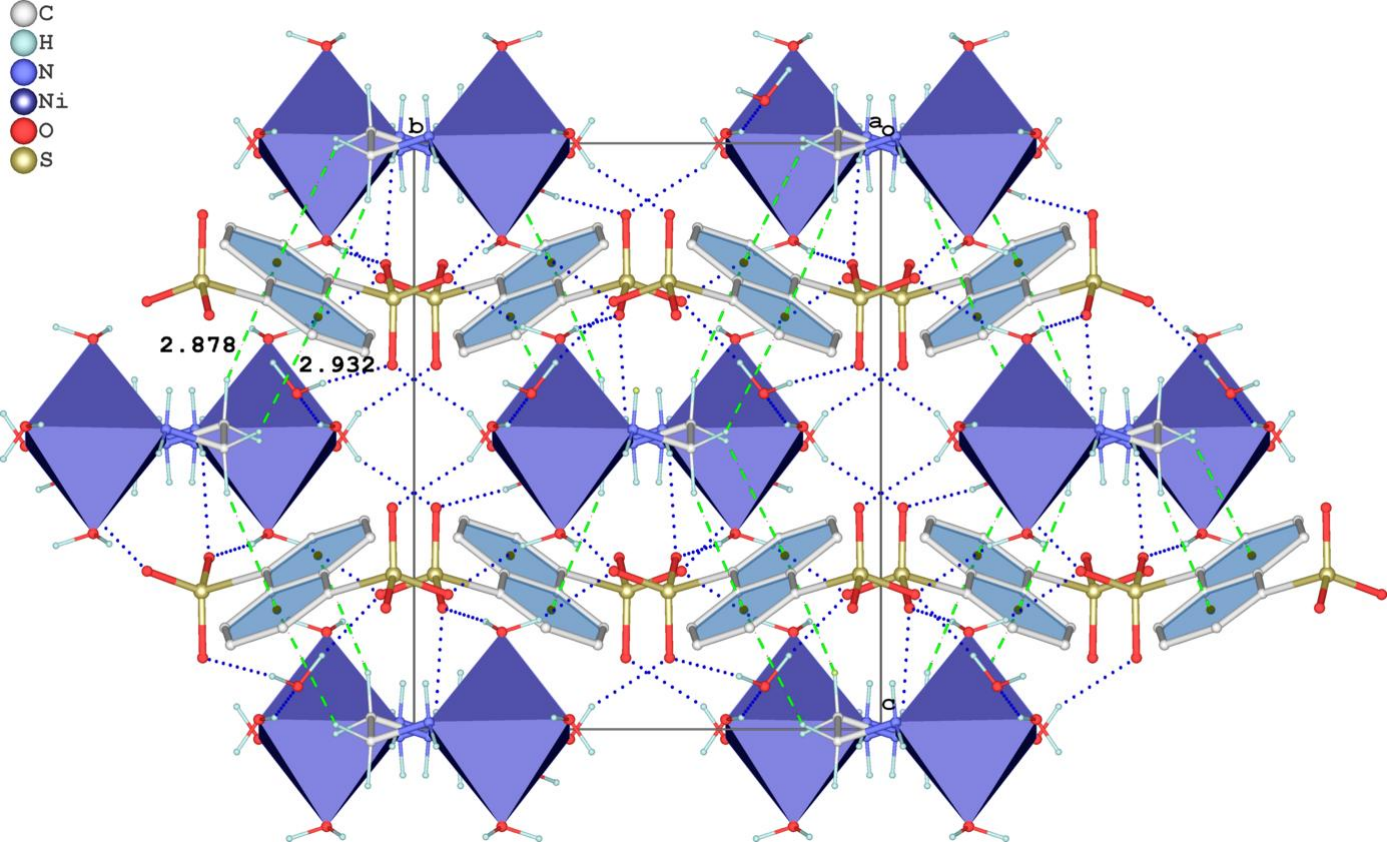
The crystal packing of the title salt in a view along [100]. O—H⋯O and N—H⋯O hydrogen bonds are shown as dashed blue lines, and C—H⋯π inter­actions as dashed green lines. The coordination polyhedron around Ni^II^ is given in the polyhedral representation.

In the crystal, the [Ni(en)(H_2_O)_4_]^2+^ cation, the NDS^2−^ anion, and the water mol­ecules are associated *via* classical O—H⋯O and N—H⋯O hydrogen bonds (Table 1 ▸). Each [Ni(en)(H_2_O)_4_]^2+^ cation forms four N—H⋯O and eight O—H⋯O hydrogen bonds with six neighboring organic anions and two water mol­ecules of crystallization. The four aqua and the en ligands in the cation participate exclusively as hydrogen-bonding donor groups (Fig. 2 ▸). All six acceptor O atoms of the SO_3_^−^ groups of the NDS^2−^ anions participate as double acceptor atoms. It should be noted that the water mol­ecule of crystallization (O3*W*) is involved in three hydrogen-bonding inter­actions: two as a donor group with two sulfonate O atoms from two different NDS^2−^ anions as acceptor atoms, and one as an acceptor group for a hydrogen bond with an aqua ligand. Next to Coulombic inter­actions, these inter­molecular inter­actions connect the mol­ecular building units into the three-dimensional supra­molecular structure, as depicted in Fig. 2 ▸. As a result of the steric hindrance caused by the sulfonate group, the nearest centroid distance between the naphthalene rings is 6.773 (2) Å. There are four notable C—H⋯π inter­actions between the methyl­ene groups of the en ligands and the naphthalene rings of the NDS^2−^ anions (Table 1 ▸, Fig. 2 ▸).

## Synthesis and crystallization

The commercially available starting materials were used without further purification. Ethane-1,2-di­amine (0.06 g, 1.00 mmol) was added slowly to an aqueous solution of NiSO_4_·7H_2_O (0.28 g, 1.00 mmol), and disodium naphthalene-1,5-di­sulfonate (0.33 g, 1.00 mmol) was added to the resulting clear deep-blue solution. The resulting solution was set out in an open beaker at room temperature. After 7 d, block-like green crystals were obtained in 60% yield (based on Ni). Elemental analysis calculated (%) for C_12_H_26_N_2_NiO_12_S_2_: C, 28.09; H, 5.11; N, 5.46; S, 12.50, found: C, 28.01; H, 5.06; N, 5.37; S, 12.43.

## Refinement

Crystal data, data collection and structure refinement details are summarized in Table 2 ▸. Hydrogen atoms attached to nitro­gen and those of the water mol­ecules were located in a difference-Fourier map and refined with bond-length restraints of 0.89 (1) and 0.85 (1) Å, respectively.

## Supplementary Material

Crystal structure: contains datablock(s) I. DOI: 10.1107/S2414314623010325/wm4202sup1.cif

Structure factors: contains datablock(s) I. DOI: 10.1107/S2414314623010325/wm4202Isup2.hkl

CCDC reference: 2311309

Additional supporting information:  crystallographic information; 3D view; checkCIF report

## Figures and Tables

**Table 1:** Hydrogen-bond geometry (Å, °) *Cg*1 and *Cg*2 are the centroids of the C1–C5/C5′ and C1′–C5′/C5 rings, respectively, where primed atoms are related by the symmetry operation 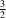 − *x*, 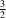 − *y*, 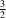 − *z*.

*D*—H⋯*A*	*D*—H	H⋯*A*	*D*⋯*A*	*D*—H⋯*A*
O1*W*—H1*WA*⋯O1^i^	0.85 (1)	1.99 (1)	2.8329 (19)	171 (3)
O1*W*—H1*WB*⋯O3*W*	0.85 (1)	1.85 (1)	2.692 (2)	176 (3)
O2*W*—H2*WA*⋯O3^i^	0.84 (1)	2.06 (1)	2.8670 (19)	160 (3)
O2*W*—H2*WB*⋯O2^ii^	0.85 (1)	1.99 (1)	2.830 (2)	168 (3)
N1—H1*A*⋯O3^iii^	0.88 (1)	2.44 (1)	3.274 (2)	159 (2)
N1—H1*B*⋯O2^iv^	0.89 (1)	2.36 (2)	3.079 (2)	139 (2)
O3*W*—H3*WA*⋯O3	0.85 (1)	1.97 (1)	2.794 (2)	164 (3)
O3*W*—H3*WB*⋯O1^v^	0.84 (1)	2.11 (1)	2.950 (2)	175 (3)
C6—H6*A*⋯*Cg*1^iii^	0.97	2.93	3.755 (2)	143
C6—H6*A*⋯*Cg*2^vi^	0.97	2.93	3.755 (2)	143
C6—H6*B*⋯*Cg*1^vii^	0.97	2.88	3.781 (2)	155
C6—H6*B*⋯*Cg*2	0.97	2.88	3.781 (2)	155

Symmetry codes: (i) 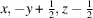 ; (ii) 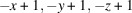 ; (iii) 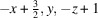 ; (iv) 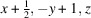 ; (v) 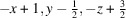 ; (vi) 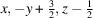 ; (vii) 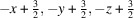 .

**Table 2:** Experimental details

Crystal data	
Chemical formula	[Ni(C_2_H_8_N_2_)(H_2_O)_4_](C_10_H_6_O_6_S_2_)·2H_2_O
*M*_r_	513.18
Crystal system, space group	Monoclinic, *I*2/*a*
Temperature (K)	291
*a*, *b*, *c* (Å)	15.4103 (3), 10.1338 (2), 13.4284 (2)
β (°)	108.692 (2)
*V* (Å^3^)	1986.44 (7)
*Z*	4
Radiation type	Cu *K*α
μ (mm^−1^)	3.99
Crystal size (mm)	0.28 × 0.24 × 0.2

Data collection	
Diffractometer	XtaLAB Synergy, Single source at home/near, HyPix3000
Absorption correction	Multi-scan (*CrysAlis PRO*; Rigaku OD, 2022 ▸)
*T*_min_, *T*_max_	0.419, 1.000
No. of measured, independent and observed [*I* > 2σ(*I*)] reflections	10963, 1926, 1881
*R*_int_	0.037
(sin θ/λ)_max_ (Å^−1^)	0.615

Refinement	
*R*[*F* ^2^ > 2σ(*F* ^2^)], *wR*(*F* ^2^), *S*	0.030, 0.085, 1.05
No. of reflections	1926
No. of parameters	165
No. of restraints	8
H-atom treatment	H atoms treated by a mixture of independent and constrained refinement
Δρ_max_, Δρ_min_ (e Å^−3^)	0.40, −0.33

Computer programs: *CrysAlis PRO* (Rigaku OD, 2022 ▸), *SHELXT* (Sheldrick, 2015*a* ▸), *SHELXL* (Sheldrick, 2015*b* ▸), *OLEX2* (Dolomanov *et al.*, 2009 ▸), and*pubCIF* (Westrip, 2010 ▸).

## full crystallographic data

### Crystal data

**Table:**

[Ni(C_2_H_8_N_2_)(H_2_O)_4_](C_10_H_6_O_6_S_2_)·2H_2_O	*F*(000) = 1072
*M**_r_* = 513.18	*D*_x_ = 1.716 Mg m^−^^3^
Monoclinic, *I*2/*a*	Cu *K*α radiation, λ = 1.54184 Å
*a* = 15.4103 (3) Å	Cell parameters from 8478 reflections
*b* = 10.1338 (2) Å	θ = 3.8–71.3°
*c* = 13.4284 (2) Å	µ = 3.99 mm^−^^1^
β = 108.692 (2)°	*T* = 291 K
*V* = 1986.44 (7) Å^3^	Block, light green
*Z* = 4	0.28 × 0.24 × 0.2 mm

### Data collection

**Table:**

XtaLAB Synergy, Single source at home/near, HyPix3000 diffractometer	1926 independent reflections
Radiation source: micro-focus sealed X-ray tube, PhotonJet (Cu) X-ray Source	1881 reflections with *I* > 2σ(*I*)
Mirror monochromator	*R*_int_ = 0.037
Detector resolution: 10.0000 pixels mm^-1^	θ_max_ = 71.5°, θ_min_ = 3.8°
ω scans	*h* = −18→18
Absorption correction: multi-scan (CrysAlisPro; Rigaku OD, 2022)	*k* = −11→12
*T*_min_ = 0.419, *T*_max_ = 1.000	*l* = −15→16
10963 measured reflections	

### Refinement

**Table:**

Refinement on *F*^2^	Hydrogen site location: mixed
Least-squares matrix: full	H atoms treated by a mixture of independent and constrained refinement
*R*[*F*^2^ > 2σ(*F*^2^)] = 0.030	*w* = 1/[σ^2^(*F*_o_^2^) + (0.0539*P*)^2^ + 1.9301*P*] where *P* = (*F*_o_^2^ + 2*F*_c_^2^)/3
*wR*(*F*^2^) = 0.085	(Δ/σ)_max_ < 0.001
*S* = 1.05	Δρ_max_ = 0.40 e Å^−^^3^
1926 reflections	Δρ_min_ = −0.33 e Å^−^^3^
165 parameters	Extinction correction: SHELXL (Sheldrick, 2015b), Fc^*^=kFc[1+0.001xFc^2^λ^3^/sin(2θ)]^-1/4^
8 restraints	Extinction coefficient: 0.00087 (12)

### Special details

**Table:**

Geometry. All esds (except the esd in the dihedral angle between two l.s. planes) are estimated using the full covariance matrix. The cell esds are taken into account individually in the estimation of esds in distances, angles and torsion angles; correlations between esds in cell parameters are only used when they are defined by crystal symmetry. An approximate (isotropic) treatment of cell esds is used for estimating esds involving l.s. planes.

### Fractional atomic coordinates and isotropic or equivalent isotropic displacement parameters (Å^2^)

**Table:**

	*x*	*y*	*z*	*U*_iso_*/*U*_eq_	
Ni1	0.750000	0.31595 (4)	0.500000	0.02364 (16)	
O1W	0.66391 (10)	0.16745 (13)	0.51659 (11)	0.0345 (3)	
H1WA	0.6474 (19)	0.106 (2)	0.4714 (17)	0.055 (8)*	
H1WB	0.6172 (15)	0.191 (3)	0.532 (3)	0.069 (10)*	
O2W	0.69189 (10)	0.31220 (13)	0.33422 (11)	0.0341 (3)	
H2WA	0.6830 (19)	0.2367 (15)	0.306 (2)	0.051 (7)*	
H2WB	0.6401 (11)	0.350 (3)	0.312 (2)	0.059 (8)*	
N1	0.83603 (11)	0.46867 (16)	0.48778 (13)	0.0301 (3)	
H1A	0.8373 (19)	0.473 (3)	0.4228 (11)	0.048 (7)*	
H1B	0.8942 (9)	0.459 (2)	0.5276 (18)	0.050 (7)*	
C6	0.80179 (13)	0.59247 (19)	0.52035 (16)	0.0363 (4)	
H6A	0.825144	0.667734	0.492234	0.044*	
H6B	0.822909	0.599187	0.596421	0.044*	
S1	0.57628 (3)	0.54449 (4)	0.76422 (3)	0.02430 (16)	
O1	0.59637 (9)	0.54691 (13)	0.87797 (10)	0.0337 (3)	
O2	0.47965 (9)	0.56091 (14)	0.70702 (11)	0.0369 (3)	
O3	0.61490 (9)	0.42790 (12)	0.73007 (11)	0.0343 (3)	
C1	0.63244 (11)	0.68365 (16)	0.73175 (13)	0.0231 (3)	
C2	0.57843 (12)	0.78162 (18)	0.67353 (14)	0.0281 (4)	
H2	0.514988	0.774290	0.653357	0.034*	
C3	0.61849 (12)	0.89311 (18)	0.64417 (14)	0.0301 (4)	
H3	0.581294	0.959325	0.604714	0.036*	
C4	0.71119 (12)	0.90538 (17)	0.67283 (14)	0.0274 (4)	
H4	0.736531	0.979213	0.651599	0.033*	
C5	0.76989 (12)	0.80698 (15)	0.73475 (13)	0.0224 (3)	
O3W	0.52056 (11)	0.25077 (18)	0.57325 (13)	0.0495 (4)	
H3WA	0.540 (2)	0.304 (2)	0.6243 (16)	0.062 (9)*	
H3WB	0.485 (2)	0.196 (3)	0.588 (3)	0.076 (11)*	

### Atomic displacement parameters (Å^2^)

**Table:**

	*U*^11^	*U*^22^	*U*^33^	*U*^12^	*U*^13^	*U*^23^
Ni1	0.0186 (2)	0.0240 (2)	0.0265 (2)	0.000	0.00471 (17)	0.000
O1W	0.0315 (7)	0.0306 (7)	0.0426 (8)	−0.0085 (5)	0.0135 (6)	−0.0048 (6)
O2W	0.0309 (7)	0.0348 (7)	0.0307 (7)	0.0010 (6)	0.0017 (6)	−0.0023 (5)
N1	0.0231 (7)	0.0330 (8)	0.0338 (8)	−0.0030 (6)	0.0086 (6)	0.0004 (6)
C6	0.0348 (11)	0.0275 (9)	0.0436 (10)	−0.0046 (8)	0.0085 (8)	−0.0027 (8)
S1	0.0179 (2)	0.0253 (2)	0.0283 (2)	−0.00158 (14)	0.00546 (17)	0.00269 (14)
O1	0.0335 (7)	0.0381 (7)	0.0309 (7)	0.0009 (5)	0.0123 (6)	0.0064 (5)
O2	0.0184 (7)	0.0412 (7)	0.0459 (8)	−0.0040 (5)	0.0030 (6)	0.0088 (6)
O3	0.0322 (7)	0.0258 (6)	0.0431 (7)	−0.0022 (5)	0.0097 (6)	−0.0036 (5)
C1	0.0185 (8)	0.0247 (8)	0.0249 (8)	−0.0009 (6)	0.0053 (6)	0.0003 (6)
C2	0.0181 (8)	0.0308 (9)	0.0324 (9)	0.0023 (7)	0.0039 (7)	0.0040 (7)
C3	0.0220 (8)	0.0281 (9)	0.0360 (9)	0.0067 (7)	0.0034 (7)	0.0099 (7)
C4	0.0235 (8)	0.0243 (8)	0.0325 (8)	0.0008 (6)	0.0064 (7)	0.0060 (7)
C5	0.0197 (8)	0.0232 (8)	0.0230 (8)	0.0004 (6)	0.0049 (6)	−0.0002 (6)
O3W	0.0438 (9)	0.0612 (10)	0.0485 (9)	−0.0163 (8)	0.0219 (7)	−0.0165 (8)

### Geometric parameters (Å, º)

**Table:**

Ni1—O1W^i^	2.0648 (14)	S1—O1	1.4583 (14)
Ni1—O1W	2.0648 (14)	S1—O2	1.4498 (13)
Ni1—O2W	2.1170 (13)	S1—O3	1.4612 (14)
Ni1—O2W^i^	2.1170 (13)	S1—C1	1.7803 (17)
Ni1—N1	2.0782 (16)	C1—C2	1.368 (2)
Ni1—N1^i^	2.0783 (15)	C1—C5^ii^	1.429 (2)
O1W—H1WA	0.847 (10)	C2—H2	0.9300
O1W—H1WB	0.846 (10)	C2—C3	1.403 (3)
O2W—H2WA	0.844 (10)	C3—H3	0.9300
O2W—H2WB	0.849 (10)	C3—C4	1.361 (2)
N1—H1A	0.880 (10)	C4—H4	0.9300
N1—H1B	0.889 (10)	C4—C5	1.422 (2)
N1—C6	1.480 (2)	C5—C5^ii^	1.428 (3)
C6—C6^i^	1.512 (4)	O3W—H3WA	0.846 (10)
C6—H6A	0.9700	O3W—H3WB	0.844 (10)
C6—H6B	0.9700		
			
O1W^i^—Ni1—O1W	86.43 (8)	N1—C6—H6A	109.9
O1W^i^—Ni1—O2W	86.76 (6)	N1—C6—H6B	109.9
O1W—Ni1—O2W	91.75 (6)	C6^i^—C6—H6A	109.9
O1W^i^—Ni1—O2W^i^	91.74 (6)	C6^i^—C6—H6B	109.9
O1W—Ni1—O2W^i^	86.76 (6)	H6A—C6—H6B	108.3
O1W^i^—Ni1—N1	94.94 (6)	O1—S1—O3	111.79 (8)
O1W^i^—Ni1—N1^i^	178.02 (6)	O1—S1—C1	106.65 (8)
O1W—Ni1—N1	178.02 (6)	O2—S1—O1	112.96 (8)
O1W—Ni1—N1^i^	94.94 (6)	O2—S1—O3	112.27 (8)
O2W^i^—Ni1—O2W	177.95 (8)	O2—S1—C1	106.06 (8)
N1—Ni1—O2W	89.77 (6)	O3—S1—C1	106.55 (8)
N1—Ni1—O2W^i^	91.76 (6)	C2—C1—S1	117.40 (13)
N1^i^—Ni1—O2W^i^	89.77 (6)	C2—C1—C5^ii^	121.20 (15)
N1^i^—Ni1—O2W	91.77 (6)	C5^ii^—C1—S1	121.41 (12)
N1—Ni1—N1^i^	83.73 (9)	C1—C2—H2	119.9
Ni1—O1W—H1WA	120.8 (19)	C1—C2—C3	120.19 (16)
Ni1—O1W—H1WB	117 (2)	C3—C2—H2	119.9
H1WA—O1W—H1WB	107 (3)	C2—C3—H3	119.6
Ni1—O2W—H2WA	116.0 (19)	C4—C3—C2	120.71 (16)
Ni1—O2W—H2WB	113 (2)	C4—C3—H3	119.6
H2WA—O2W—H2WB	105 (3)	C3—C4—H4	119.5
Ni1—N1—H1A	109.5 (17)	C3—C4—C5	120.97 (16)
Ni1—N1—H1B	114.7 (17)	C5—C4—H4	119.5
H1A—N1—H1B	105 (2)	C4—C5—C1^ii^	123.08 (15)
C6—N1—Ni1	108.12 (12)	C4—C5—C5^ii^	118.94 (19)
C6—N1—H1A	112.0 (17)	C5^ii^—C5—C1^ii^	117.98 (18)
C6—N1—H1B	107.4 (17)	H3WA—O3W—H3WB	108 (3)
N1—C6—C6^i^	109.15 (12)		
			
Ni1—N1—C6—C6^i^	−38.8 (2)	O3—S1—C1—C5^ii^	−53.25 (16)
S1—C1—C2—C3	−179.17 (14)	C1—C2—C3—C4	0.1 (3)
O1—S1—C1—C2	−113.59 (15)	C2—C3—C4—C5	−1.1 (3)
O1—S1—C1—C5^ii^	66.29 (15)	C3—C4—C5—C1^ii^	−178.96 (17)
O2—S1—C1—C2	7.07 (16)	C3—C4—C5—C5^ii^	1.1 (3)
O2—S1—C1—C5^ii^	−173.05 (14)	C5^ii^—C1—C2—C3	1.0 (3)
O3—S1—C1—C2	126.87 (14)		

Symmetry codes: (i) −*x*+3/2, *y*, −*z*+1; (ii) −*x*+3/2, −*y*+3/2, −*z*+3/2.

### Hydrogen-bond geometry (Å, º)

*Cg*1 and *Cg*2 are the centroids of the C1–C5/C5' and C1'–C5'/C5 rings, respectively, where primed atoms are related by the symmetry operation 3/2 - *x*, 3/2 - *y*, 3/2 - *z*.

**Table:**

*D*—H···*A*	*D*—H	H···*A*	*D*···*A*	*D*—H···*A*
O1*W*—H1*WA*···O1^iii^	0.85 (1)	1.99 (1)	2.8329 (19)	171 (3)
O1*W*—H1*WB*···O3*W*	0.85 (1)	1.85 (1)	2.692 (2)	176 (3)
O2*W*—H2*WA*···O3^iii^	0.84 (1)	2.06 (1)	2.8670 (19)	160 (3)
O2*W*—H2*WB*···O2^iv^	0.85 (1)	1.99 (1)	2.830 (2)	168 (3)
N1—H1*A*···O3^i^	0.88 (1)	2.44 (1)	3.274 (2)	159 (2)
N1—H1*B*···O2^v^	0.89 (1)	2.36 (2)	3.079 (2)	139 (2)
O3*W*—H3*WA*···O3	0.85 (1)	1.97 (1)	2.794 (2)	164 (3)
O3*W*—H3*WB*···O1^vi^	0.84 (1)	2.11 (1)	2.950 (2)	175 (3)
C6—H6*A*···*Cg*1^i^	0.97	2.93	3.755 (2)	143
C6—H6*A*···*Cg*2^vii^	0.97	2.93	3.755 (2)	143
C6—H6*B*···*Cg*1^ii^	0.97	2.88	3.781 (2)	155
C6—H6*B*···*Cg*2	0.97	2.88	3.781 (2)	155

Symmetry codes: (i) −*x*+3/2, *y*, −*z*+1; (ii) −*x*+3/2, −*y*+3/2, −*z*+3/2; (iii) *x*, −*y*+1/2, *z*−1/2; (iv) −*x*+1, −*y*+1, −*z*+1; (v) *x*+1/2, −*y*+1, *z*; (vi) −*x*+1, *y*−1/2, −*z*+3/2; (vii) *x*, −*y*+3/2, *z*−1/2.
